# Central Neural Correlates During Inhibitory Control in Lifelong Premature Ejaculation Patients

**DOI:** 10.3389/fnhum.2018.00206

**Published:** 2018-05-22

**Authors:** Xuejuan Yang, Ming Gao, Lan Zhang, Lin Liu, Peng Liu, Jinbo Sun, Yibin Xi, Hong Yin, Wei Qin

**Affiliations:** ^1^Engineering Research Center of Molecular and Neuro Imaging of the Ministry of Education, School of Life Science and Technology, Xidian University, Xi’an, China; ^2^Department of Urology, Xijing Hospital, The Fourth Military Medical University, Xi’an, China; ^3^The ART Center, The Northwest Women’s and Children’s Hospital, Xi’an, China; ^4^Department of Radiology, Xijing Hospital, The Fourth Military Medical University, Xi’an, China

**Keywords:** lifelong premature ejaculation, stop signal task, inhibitory control, neural activation, functional connectivity

## Abstract

Lifelong premature ejaculation (LPE) is a common male sexual dysfunction. Lack of active control for rapid ejaculation brought great distress to sexual harmony and even fertility. Previous neurophysiology studies revealed an ejaculation-related control mechanism in the brain. However, it remains unclear whether this inhibitory network is altered in LPE patients. The present study investigated the central inhibitory network function of LPE patients by using stop signal task (SST)-related functional magnetic resonance imaging (fMRI) and resting-state functional connectivity (FC) analysis. The results showed no difference in task-related behavioral performance or neural activation during response inhibition between LPE patients and controls. However, LPE patients showed a significantly different correlation pattern between the stop signal reaction time (SSRT) and left inferior frontal gyrus (IFG) activation during successful inhibition, in which a typical negative correlation between SSRT and the activation was completely disappeared in patients. In addition, using the left IFG as a seed, patients showed weaker FC between the seed and two areas (left dentate nucleus (DN) and right frontal pole) compared with controls. These data suggest that LPE patients have an abnormal brain control network, which may contribute to the reduced central control of rapid ejaculation. This study provides new insights into the neural mechanism of LPE involving the central inhibitory network, which may offer an underlying intervention target for future treatment.

## Introduction

Lifelong premature ejaculation (LPE) is a common male sexual dysfunction, with a prevalence of 3% in the Chinese population (Althof et al., 2014). In the latest edition of the Diagnostic and Statistical Manual of Mental disorders (DSM-5™), PE is defined as “a persistent or recurrent pattern of ejaculation occurring during partnered sexual activity within approximately 1 min following vaginal penetration and before the individual wishes it”, the LPE is specified as “the disturbance has been present since the individual became sexually active” (American Psychiatric Association, 2013). According to this definition, congenital disability of the inhibitory control of rapid ejaculation (<1 min) impulsivity is a typical feature of LPE. Previous animal studies have proposed a central control mechanism for ejaculation inhibition (Wolters and Hellstrom, 2006), and LPE had been suggested to be comparable with impulsive control disorders because of similarities in terms of loss of behavioral control and involvement of the central serotonergic and dopaminergic systems (Ozdemir, 2012). Thus, LPE patients may have abnormalities in the central neural circuitry mediating inhibitory control, although this has not been specifically examined.

A recent neuroimaging study reported a different pattern of brain activation in response to erotic stimulation in LPE patients, in which the abnormal areas included the inferior frontal gyrus (IFG), middle temporal gyrus and supplementary motor area (SMA; Zhang et al., 2017). Coincidentally, the IFG and SMA were also the core locus of the motor inhibitory control network (Aron and Poldrack, 2006; Swann et al., 2012; Aron et al., 2014; Jha et al., 2015). Moreover, the central dopamine neurotransmitter system, an important regulator of response inhibitory control (Robertson et al., 2015), is an alternative pharmacological target in clinical treatment of LPE (Peeters and Giuliano, 2008), while serotonin reuptake inhibitors were reported to delay vaginal ejaculation in LPE (Castiglione et al., 2016) and increase stopping success rate in inhibition tasks (Overtoom et al., 2003). Thus, in consideration of the overlapping brain centers and neurotransmitter mechanisms shared by LPE and inhibition control (such as motor inhibitory control), we speculate that LPE patients may exhibit impaired inhibition-related brain activity in response to inhibitory stimuli. A better understanding of the neural correlates of response control in LPE patients may provide novel information on the neurobiological etiology of LPE and provide new potential treatment options.

The stop signal task (SST) is a classical paradigm used to evaluate the ability of motor control and can reliably detect the neural circuit for response inhibition (Verbruggen and Logan, 2008). Psychopathologists have used SST to study inhibitory deficits in a variety of diseases, including Parkinson’s disease, attention deficit-hyperactivity disorder, compulsive disorders, substance-abuse disorders and stress disorder (Chamberlain et al., 2007; Li et al., 2008; Galván et al., 2011; van Rooij et al., 2014; Claassen et al., 2015; van Rooij D. et al., 2015; van Rooij S. J. et al., 2015; Vriend et al., 2015). Therefore, in the present study, we used SST to investigate the brain inhibition response in LPE patients. By using event-related functional magnetic resonance imaging (fMRI), we compared the inhibition-related neural activation between LPE patients and healthy controls (HCs) and examined the correlations of neural activation in inhibition response with the stop signal reaction time (SSRT), an indicator of control capacity and several clinical indexes, including the rating of PE diagnostic tool (PEDT) and intravaginal ejaculatory latency time (IELT). Differences in integration within the entire brain related to inhibition control between LPE patients and HCs were also evaluated by functional connectivity (FC) analysis.

## Materials and Methods

### Participants

All participants were heterosexual right-handed male Chinese volunteers. LPE patients were recruited from out-patients in the Northwest Women’s and Children’s Hospital, China. LPE diagnoses were based on the International Society for Sexual Medicine guidelines and DSM-5™ (American Psychiatric Association, 2013) and the International Society for Sexual Medicine’s guidelines for the diagnosis and treatment of PE (Althof et al., 2010). All participants underwent history taking and physical examination. Each patient always had an IELT within 1 min. The PEDT score of each LPE patient was >11, but <5 for each control. All participants were non-smokers, with an International Index of Erectile Function (IIEF) score >21. Subjects with psychiatric disorders were excluded, and no participants reported a medical or neurological disorder and no alcohol, nicotine or drug abuse. Patients did not receive any treatment at least 2 weeks before the experiment. There were no differences in intelligence quotient between groups according to the Chinese Revised Wechsler Adult Intelligence Scale. Written informed consent was obtained from all study participants. Research procedures were approved by the ethical committee of the Northwest Women’s and Children’s Hospital in China and were conducted in accordance with the Code of Ethics of the World Medical Association (Declaration of Helsinki).

On the day of the experiment, the T1 data and resting-state fMRI (rs-fMRI) data were acquired first, followed by SST-related fMRI scans.

### Behavioral Task

The SST used in the present study was similar to that reported (Logan, 1994; Aron and Poldrack, 2006). Briefly, two types of tasks were included: 75% of go trial and 25% of stop trial. During the go task, subjects were asked to press a certain key as fast and accurately as possible when the stimulus was displayed on a screen. Each stimulus in the go trial was presented for 1000 ms. During the stop task, subjects were required to inhibit the response by pressing any key when the stop signal (white arrow changed to blue) was displayed. The stop signal was presented at a particular delay (stop signal delay, SSD) that was dynamical adjusted according to the last stop task performance. If the stop trial was finished successfully, the SSD was increased by 50 ms, or else decreased by 50 ms. This method provides a rate of inhibition success in the stop trial of approximately 50%, and thus controls the difficulty level across subjects and eliminates practice effects.

During scanning, participants were asked to complete two runs, each containing two blocks with 36 go trials and 12 stop trials per block. Each block lasted for 4.5 min. The arrangement of the SSD was derived from two staircases that started with SSD values of 250 and 350 ms, respectively. The order of the staircases was randomized. Null events were inserted between each go or stop trial with a duration ranging from 0.5 s to 4 s (1 s average, exponential distribution). SST training was performed immediately before scanning for each participant.

### Scanning Acquisition

Imaging data were collected using a 3T MRI system (EXCITE, General Electric, Milwaukee, WC, USA) at a local hospital. A standard birdcage head coil was used, along with restraining foam pads, to minimize head motion and to reduce scanner noise. High-resolution T1-weighted images were acquired (repetition time = 8.2 ms; echo time = 3.2 ms; flip angle = 12°; field of view = 256 × 256 mm^2^; data matrix = 256 × 256; in-plane resolution = 1 × 1 mm^2^; slice thickness = 1 mm). Head movement was restricted using foam padding. Whole brain fMRI images were obtained using the gradient echo (repetition time = 2000 ms, echo time = 30 ms; field of view = 240 × 240 mm^2^; matrix size = 64 × 64; flip angle = 90°; in-plane resolution = 3.75 × 3.75 mm^2^; slice thickness = 3.5 mm with no gaps; 45 axial slices). A total of 210 volumes were acquired in the rs-fMRI scan, and 270 volumes were acquired in each task-fMRI scan.

### Behavioral Data Analyses

We used the quantile method to determine SSTR (Band et al., 2003). All reaction times (RTs) on the correct go trials were arranged in ascending order. The RT corresponding to the proportion of failed inhibition was selected as the quantile RT. SSRT was estimated by subtracting the average SSD from the quantile RT.

Subjects with the following SST performance were excluded from analysis: (1) accuracy of the stop trials <25% or >75%; (2) percentage of correct go trial <60%; (3) percentage of go trial response to error >10%; (4) percentage of go trial with missing >20%; and (5) SSRT negative or <50 ms (Congdon et al., 2010; Thakkar et al., 2014). The demographics and SST variables were compared between the LPE and control groups using independent samples *t*-tests.

### Imaging Data Analyses

#### Preprocessing

We used SPM12^1^ and the MATLAB 2012a software package (MathWorks, Natick, MA, USA) to process fMRI data. Pre-processing of fMRI data included slice-timing correction, realignment, spatial normalization and smoothing. First, all volumes were aligned with the first volume to correct head movement (translation more than 1.0 mm or rotation more than 1.0° being excluded). Second, we coregistered the images to the Montreal Neurological Institute (MNI) space and resampled at 3 × 3 × 3 mm^3^. Third, we used a 6-mm full width at half maximum Gaussian kernel for smoothing.

#### One Sample *T*-Test

The events of the successful go and successful inhibition were modeled using a standard general linear model. The event was modeled when the arrow was stimulated. To improve statistical sensitivity, we used the time derivative as a covariate without interest. Empty events were not explicitly modeled, and thus constituted an implicit baseline (Aron and Poldrack, 2006; Galván et al., 2011). For each subject and each scan, we calculated the contrast images of successful inhibition > successful go. The active regions of this contrast in LPE patients and controls were analyzed at the whole brain level using a one sample *t*-test. Age was included as a covariate. The threshold was set at *P* < 0.05 (corrected for false discovery rate (FDR)) rate.

#### Two-Sample *T*-Test

Using a two-sample *t*-test, we calculated the difference in brain activation of the contrast (successful inhibition > successful go) between controls and LPE patients at the whole brain level. Correlation analysis was performed between clinical parameters and significant brain activation when there were neural differences between the groups. Age was included as a covariate. The significance threshold was also set at *P* < 0.05 (FDR corrected).

#### Correlation Analysis and Cross Validation

Variance analysis was used to examine the interaction effect between group and brain activation in the contrast of successful inhibition > successful go in predicting SSRT. Age was included as a covariate (*P* < 0.001 uncorrected, cluster size *P* < 0.05, FDR corrected). For brain activation that showed an interactive correlation with SSRT by the group, *post hoc* analysis was performed to examine the different predictive patterns in the two groups.

To verify the predictive power, we conducted a 1000 times 3-fold-cross-validation test respectively for both groups. First, all subjects were randomly divided into three equal parts (two of them as a training set, one as a test set), and we repeated three iterations for all training-testing combinations. In the three iterations, the training set was used to obtain the regression value of the SSRT though the brain image activation value. The activation value of the brain image of the test set was then inputted into the obtained equation, and the correlation of the real value with the predicted value was calculated, thus providing three correlation values, that were then averaged to obtain a mean correlation value. Finally, to stabilize the final correlation value, the above steps were repeated 1000 times to obtain a distribution of 1000 correlation values. If 95% of the correlation values was >0, then the prediction was reliable (Kim, 2009; Jin et al., 2017).

#### Resting-State FC Analysis

To further analyze the inhibition brain network of patients during the resting-state, the brain areas showing a significant difference in above two sample *t*-test and correlation analysis were collected as the seed regions in order to evaluate the resting brain function between the groups. The resting-state FC analysis was performed with the CONN toolbox^2^. First, the T1 image was segmented into gray matter, white matter and cerebrospinal fluid. Gray and white matter maps were added and used as masks to create a brain-extracted version of the anatomical images. Images were then normalized to the MNI template. For rs-fMRI analysis, in order to ensure the reliability of the resting data, we removed the first four images, and then performed the slice timing correction and realignment. The corrected image was coregistered to the anatomical scan and resampled at 3 × 3 × 3 mm^3^. Outliers were detected and Gaussian smoothing performed with a 6 mm full width at half maximum Gaussian kernel. Linear regression and band-pass filtering were applied to remove unwanted motion, physiological, effects and other artifacts from the signals before computing connectivity measures. The main steps include: (1) linear detrending; (2) regressing out the six head motion parameters and their first-level derivative, the averaged cerebrospinal fluid and white matter signals, and the scrubbing signal (i.e., the outlier information of the scans) from the time series; (3) 0.01–0.1 Hz band-pass filtering. We then performed a General Linear Model weighted regression/correlation measure of the condition-specific association of the seed time series with each voxel time series of the whole brain. The *z*-map of each subject was produced using the *Fisher* r-to-z transformation to improve normality (Liu et al., 2017). Finally, FC differences between the groups (LPE vs. controls) were analyzed with the age as a covariate (voxel-wise threshold *P* < 0.001 uncorrected and *P* < 0.05 FDR corrected at cluster level).

## Results

### Demography and SST Performance

**Figure 1:**
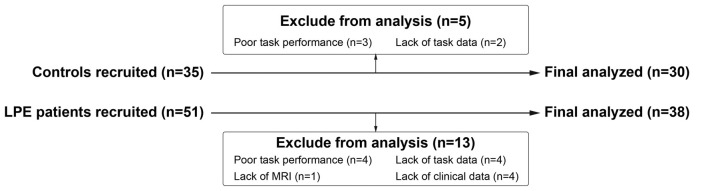
Diagram of participants recruited and excluded. MRI, Magnetic Resonance Imaging.

Thirty control subjects and 38 LPE patients were included in final analyses (Figure 1). As shown in Table 1, there were no significant differences between the LPE and control groups in age or IIEF-5 score. The average disease duration for patients was longer than 5 years. And the LPE group had a markedly shorter IELT and higher PEDT score than controls (*P* < 0.0001). For the SST task, the percentage of successful inhibition was approximately 50% in both groups, with no differences in go task and stop task performance between the groups.

**Table 1:** Demographic, clinical and stop signal task (SST) data for participants.

Group	LPE (*n* = 38)	HC (*n* = 30)
Age (years)	30.53 ± 5.06	31.33 ± 2.77
	range: 21–45	range: 24–37
IIEF-5 score	23.87 ± 0.93	24.23 ± 0.89
IELT (min)	0.62 ± 0.28	10.73 ± 6.10***
PEDT score	17.50 ± 1.96	0.80 ± 1.40***
Duration (year)	5.54 ± 3.53	0
*SST performance*		
Correct Go reaction time (ms)	561.50 ± 92.05	540.28 ± 120.61
Percentage of Go discrimination errors	0.01 ± 0.01	0.01 ± 0.02
Percentage of Go miss errors	0.01 ± 0.02	0.01 ± 0.03
Percentage of successful inhibition	0.52 ± 0.11	0.49 ± 0.15
Mean stop signal delay (ms)	267.68 ± 75.91	256.60 ± 95.88
Stop signal reaction time (ms)	277.7 ± 33.39	269.98 ± 32.64

Data were presented as mean ± SD. HC, healthy control; IELT, intravaginal ejaculatory latency time; IIEF-5, International Index of Erectile Function-5; LPE, lifelong premature ejaculation; PEDT, Premature ejaculation diagnostic tool. ***P < 0.0001 by independent samples t-test.

### Brain Activation During the Inhibition Process

**Figure 2:**
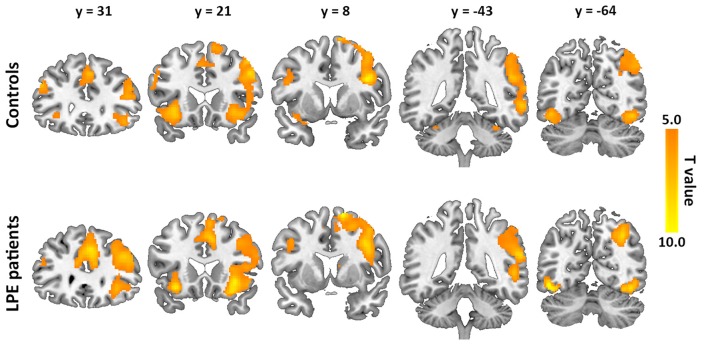
Neural activation during the inhibition process (successful inhibition > successful go contrast) in the controls and lifelong premature ejaculation (LPE) patients.

The inhibition-related neural networks in the control and LPE groups for successful inhibition > successful go contrast (*P* < 0.05) are shown in Figure 2. For both the control and LPE groups, significant activation was found in the bilateral IFG, anterior and middle cingulate cortex, insular, SMA, superior frontal gyrus and inferior occipital cortex. The right middle frontal gyrus, supramarginal gyrus and middle temporal gyrus were also activated. This activation distribution was consistent with previous studies (Bonnelle et al., 2012; van Rooij D. et al., 2015), apart from no activation in the basal ganglia region. There was no difference in inhibition-related neural activation between the groups.

### Correlation Analysis and Cross-Validation Test

**Figure 3:**
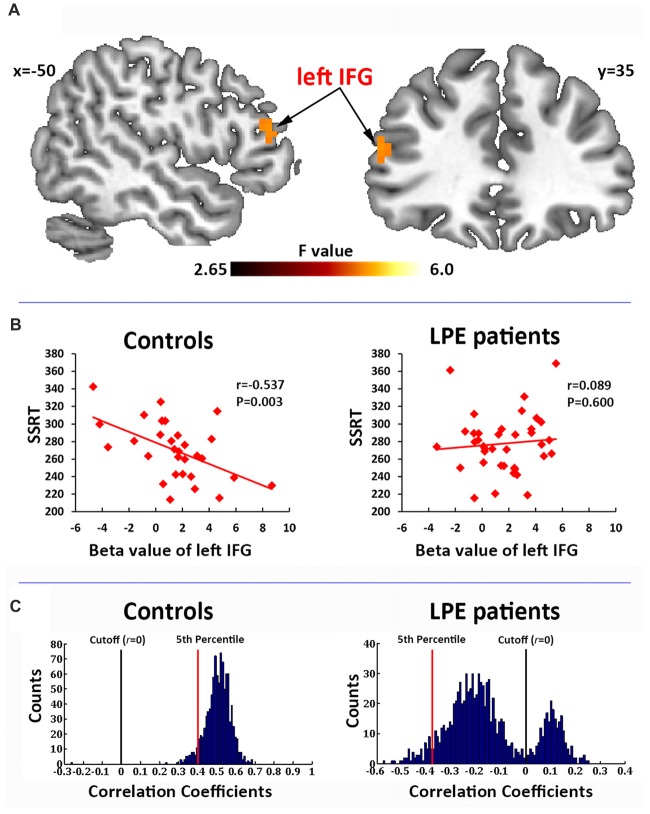
Correlation of brain activation with stop signal reaction time (SSRT). **(A)** Activation of the left inferior frontal gyrus (IFG) during the inhibition process (successful inhibition > successful go contrast) had a significant interaction with the SSRT by groups. **(B)** The beta value of the left IFG had a significant negative correlation with SSRT in the controls, but not in LPE patients. **(C)** A 1000 time three-fold cross validation of the left IFG activation predicting SSRT in controls and LPE patients. In controls, 99.8% of the *r* value between the predicted and observed SSRT was >0, while only 24.9% was >0 in LPE patients.

In the correlation analysis, a significant interaction of the left IFG (BA45, MNI coordinate: −51, 36, 18) activation by groups was found to predict SSRT performance (Figure 3A). *Post hoc* test on the mean beta averaged across significant voxels showed that the SSRT was significantly negatively correlated with left IFG activation in controls (*r* = −0.537, *P* = 0.003, Figure 3B), but not in LPE patients (*r* = 0.089, *P* = 0.600, Figure 3B).

We then used 1000 times three-fold cross validation to test the robustness of the prediction of left IFG activation on SSRT in the control and LPE groups. As shown in Figure 3C, 99.8% of the correlation coefficients (mean *r* = 0.51) between the predicted and observed SSRT value over 1000 iterations were >0 in controls, while only 24.9% of the correlation coefficients (mean *r* = −0.14) were >0 in LPE patients. These data provide further support for loss of the reliable predictive relationship of IFG activation on SSRT in LPE patients. There were no main effects of neural activation. No correlations were found between clinical data (PEDT score, IELT) and the activation of inhibition network.

### Resting-State FC Analysis

**Figure 4:**
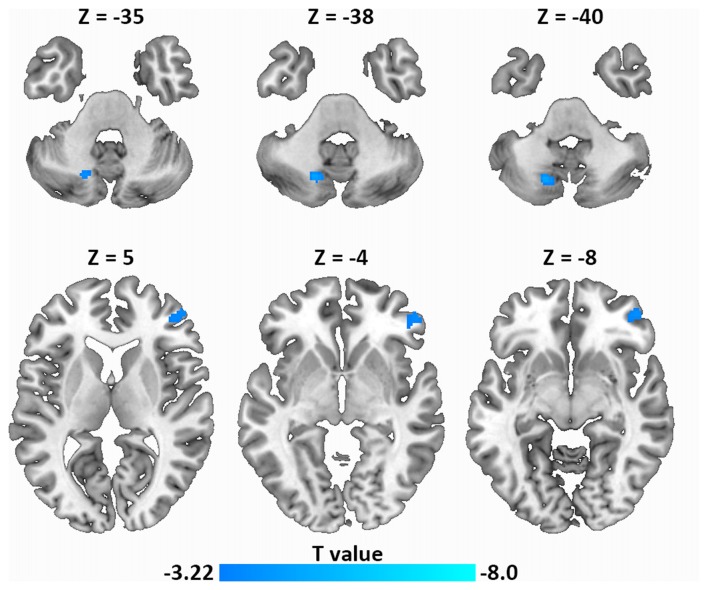
Decreased resting-state functional connectivity (FC; left IFG as a seed) in LPE patients compared with controls during resting state (*P* < 0.001, uncorrected, cluster size *P* < 0.05 false discovery rate (FDR) corrected). Top panel, left dentate nucleus (DN); Bottom panel, frontal pole.

Next, using resting-state FC analysis with the left IFG as a seed, we compared the resting-state connectivity strength in the whole brain between the two groups. As shown in Figure 4, the left IFG showed significantly decreased FC with the right frontal pole (MNI coordinate: 52, 46, −4) and the left dentate nucleus (DN) of the cerebellum (MNI coordinate: −18, −68, −40) in LPE patients compared with controls.

## Discussion

Since the use of the central dopaminergic and serotonergic systems as pharmacological targets for LPE treatment, there has been increasing interest in the neurobiological mechanisms of LPE. However, human data are still limited, except for several electroencephalography and neuroimaging studies detecting abnormal spontaneous and evoked brain activation responses to erotic stimuli, brain structure changes in PE patients (Hyun et al., 2008; Kwon et al., 2011; Guo et al., 2017; Zhang et al., 2017). Our findings provide a new understanding of the neural mechanism of LPE from an inhibition control perspective. By using the SST, a classic inhibition control task, we found that the correlation of neural activation during inhibition with SSRT was completely different in control and LPE groups. SSRT was negatively correlated with the left IFG activation in controls, but no correlation was observed in LPE patients. Moreover, the seed region (left IFG) in LPE patients had weaker resting-state FC with the left DN and right frontal pole compared with controls. This atypical association of the SSRT with neural activation, and the weaker connections related to control and sexual circuits suggests a potential abnormality in the central inhibition control network in LPE patients, although no significant differences in inhibitory neural activation or SSRT performance were found between the groups.

A number of studies have found that the IFG is a core area involved in inhibitory network responses to SST (Aron and Poldrack, 2006; Swann et al., 2012; Aron et al., 2014; Jha et al., 2015). Activation of the IFG was often negatively correlated with the SSRT; i.e., a greater IGF response predicted better inhibition capacity (smaller SSRT; Galván et al., 2011; van Rooij D. et al., 2015). Dysfunction of the IFG during inhibition response was also reported in psychological diseases such as attention deficit hyperactivity disorder, Parkinson’s disease and posttraumatic stress disorder (van Rooij et al., 2014; van Rooij D. et al., 2015; Vriend et al., 2015). Although most studies have detected the right IFG, the left IFG was also reported to play a role in inhibition control performance (Bari and Robbins, 2013). Indeed, in attention deficit hyperactivity disorder patients, a marked hypoactivation was found in the left IFG compared with HCs, with an association of the left IFG with SSRT (van Rooij D. et al., 2015). During the go/no-go task, abnormal activation in the left IFG was also found in smokers and healthy subjects, while patients with a left IFG lesion showed impaired control performance (Fassbender et al., 2004; Swick et al., 2008; Luijten et al., 2013). Even in resting-state, spontaneous activity in the left IFG was significantly correlated with SSRT (Tian et al., 2012; Lee and Hsieh, 2017). The bilateral IFG activation in response to inhibition process in both control and LPE groups also provides support that the process of control response may require the involvement of the left and right IFG together. Therefore, in the present study, the loss of correlation of left IFG activation with SSRT in LPE patients suggest a potential abnormality in the cortical control network.

Neurophysiological studies have confirmed that ejaculation is a complex and highly coordinated progressing process that depends on the synchronization of autonomic and somatic nervous system activity, which is integrated in the spinal cord and specific brain structures (Giuliano and Clement, 2005; Jannini et al., 2013). Animal studies have proposed a supraspinal level control mechanism to inhibit ejaculation, in which brain areas, such as the posteromedial bed nucleus of the stria terminalis, posterodorsal medial amygdaloid nucleus, posterodorsal preoptic nucleus and the parvicellular part of the subparafascicular thalamus produce a basal inhibitory tone on the spinal mechanism of ejaculation (Wolters and Hellstrom, 2006). However, little is known about the role of the cerebral cortex in ejaculation control. In 2003, Holstege et al. reported regional cerebral blood flow changes related to ejaculation in men (Holstege et al., 2003). In 2007, they revised their study because of some methodological flaws (Georgiadis et al., 2007), and found that regional cerebral blood flow actually decreased throughout the prefrontal cortex (including the left IFG), indicating a crucial role of the prefrontal cortex in inhibitory control over ejaculation, although this inhibitory control may expand to events occurring immediately before (sexual arousal) and/or after ejaculation (sexual satiety) because of the 120-s image span for the [^15^O]-H_2_O positron emission tomographic scan. Nevertheless, together with our present findings of an underlying abnormality of left IFG function in LPE patients in a control task (SST), these studies suggest that functional impairment of the prefrontal cortex, especially the left IFG, may be causative in LPE. Thus, the atypical association control network activation with SSRT in LPE suggests an insufficient control of left IFG on ejaculation, which may contribute to uncontrollable rapid ejaculation. Of note, a recent fMRI study revealed dysfunction of left IFG in LPE patients in response to visual sexual stimuli, and with evidence of abnormal regional homogeneity and resting FC with the bilateral SMA (also a core region in the control network; Zhang et al., 2017). These findings provide further support for the role of left IFG in the etiology of LPE.

We also evaluated the whole brain FC with the left IFG and found that the FC between the left IFG and the DN, and between the left IFG and the right frontal pole, was significantly reduced in LPE patients. Two previous neuroimaging studies on male ejaculation have confirmed a prominent role of DN in ejaculation (Holstege et al., 2003; Georgiadis et al., 2007), although this may be related to pelvic muscular contractions during intercourse (Georgiadis et al., 2006). The DN was also reported to have functional and reciprocal anatomical connections with the prefrontal area, which contributed to both motor regulation and cognitive function (Allen et al., 2005; Bernard et al., 2014). Further, there is evidence of inhibition modulation from the prefrontal cortex to the DN during sexual activity, in which behavioral disinhibition from the prefrontal area to the DN likely promotes sexual experience (Georgiadis et al., 2006). Therefore, in the present study, the decreased coherence of the left IFG and the DN in LPE patients suggests insufficient control of the IFG on the DN in sexual function. Together with the involvement of the frontal pole in the inhibition control task (Brier et al., 2010), in our present work, the unstable connection within the inhibition network (left IFG—right frontal pole) and weaker connection between the inhibition network and the neural pathways of sexual activity (left IFG—left DN) in LPE patients, may reflect a wider dysfunction of regions related to central inhibitory control of ejaculation.

There were several limitations. First, in the current work, we used the classic SST which focus on motor control, but an SST designed with erotic pictures may be more effective for detecting the capacity of inhibition control in LPE patients, although motor control (reflected in our SST) is also involved in ejaculation behavior. Second, only PEDT and IELT were used in our analysis. Other factors, such as sexual arousal level, which is often studied in LPE etiology, were not considered. Thus, other indicators of sexual physiology should be included in future studies, although the arousal dysfunction in LPE remains controversial (Rowland, 2005). Third, the absence of group difference may be related to the relatively small sample size used in our study. Thus, future studies with large numbers of patients are required to confirm our findings.

In conclusion, we provide new evidence for the neural mechanism of LPE. An SSRT-related activation difference in the control network between LPE patients and healthy subjects was found in the left IFG of the prefrontal lobe, which produced weak connections with the ejaculation-related cerebellar region and the control network. Future studies examining the role of the PFC in LPE patients will help determine the etiology of LPE and provide potential therapeutic targets for neural intervention treatments.

## Author Contributions

XY, MG, and WQ: designed the study. LZ, XY, LL, and PL: analyzed the data. MG, LL, YX, and HY: conducted the experiments. XY, HY, WQ, and JS: interpretation of data. XY and MG: wrote the article.

## Conflict of Interest Statement

The authors declare that the research was conducted in the absence of any commercial or financial relationships that could be construed as a potential conflict of interest.

## Footnotes

^1^http://www.fil.ion.ucl.ac.uk/spm

^2^https://www.nitrc.org/projects/conn/
